# The Effect of COVID-19 on College Students’ Entrepreneurial Intentions

**DOI:** 10.3389/fpsyg.2022.870705

**Published:** 2022-03-24

**Authors:** Fan Sheng, Yangyang Chen

**Affiliations:** ^1^School of Economics and Management, Harbin Engineering University, Harbin, China; ^2^School of Economics and Management, Hebei University of Technology, Tianjin, China; ^3^School of Economics and Management, Dalian University of Technology, Dalian, China; ^4^Dalian Yongjia Electronic Technology Co., Ltd., Dalian, China

**Keywords:** COVID-19, event strength, fear of failure, regulatory focus, entrepreneurial intentions

## Abstract

The new coronary pneumonia epidemic has had a tremendous impact on the world economic situation, causing a large number of enterprises to suffer from serious losses, but also bringing a large number of entrepreneurial opportunities. For college students, whether the opportunities brought by the epidemic can attract them to step into the entrepreneurial path becomes a question worthy of attention in the process of restoring economic vitality and guiding students’ employment and entrepreneurship. In this article, a mediation model was constructed and tested through 245 questionnaire data by combining event system theory, regulatory focus theory, and emotion cognitive evaluation theory. The results showed that defensive regulatory focus and fear of failure and facilitative regulatory focus and fear of failure were all able to continuously mediate the effect of event intensity of the new coronary pneumonia epidemic on the entrepreneurial intentions of college students.

## Introduction

Coronavirus disease 2019 (COVID-19) pandemic that emerged at the end of 2019 is having a profound impact on the global business environment, with a large number of enterprises suffering from severe losses and even facing bankruptcy crises, alongside a significant increase in unemployment. Ibáñez et al. (2021) argue that the pandemic will have a persistently negative effect on investment, jobs, schooling, and international trade. Furthermore, in the context of the slow global economic recovery in 2021, new COVID-19 strains such as the Delta variant have added to the economic strain. According to the International Labor Organization, 8.8% of global work time was lost in 2020, equivalent to 255 million full-time jobs. In particular, young people aged 15–24 were among the most severely affected, with an unemployment rate of 8.7%. College graduates are members of the group most affected by the pandemic and the damage caused will expose them to employment difficulties in the future, which will, thus, affect the normal order of society. However, there are always two sides to the coin. While negatively impacting society and the economy, the changes brought about by the pandemic may be beneficial to certain types of businesses. Moreover, the high level of uncertainty it creates and the subsequent elevation of new, urgent needs present unique entrepreneurial opportunities, which provide room for the creation and growth of many new businesses in various sectors. For example, online education, home entertainment, telemedicine, e-commerce, medical devices, and other industries all flourished during the pandemic (Bacq and Lumpkin, 2021; Lungu et al., 2021; Zahra, 2021). Overall, the pandemic had a more lasting impact on people’s values, jobs, lifestyles, and mindsets, in addition to their health (Davidsson et al., 2021). For college students, the restrictions on gathering, social distance, and travel affected their studies, make them more educated online, while the impact on employers indirectly affected their regular employment. Furthermore, traditional employment channels have changed, with students’ preferences moving from large corporations or government departments to start-ups and entrepreneurship (Akkermans et al., 2020; Ratten and Jones, 2021). Various studies found that, by creating, expanding, and substituting demand, the pandemic environment created a wealth of entrepreneurial opportunities and motivated people to replace regular employment with entrepreneurship (Maritz et al., 2020; Alvarez-Risco et al., 2021), which creates rich preconditions for entrepreneurship among college students. Moreover, when an individual perceives an entrepreneurial opportunity, they may favor the option of starting a business (Karimi et al., 2016).

Alvarez-Risco et al. (2021) argue that promoting entrepreneurial activities can help to combat the crisis brought about by the pandemic. Therefore, with the urgent need to restart the economy, whether students are willing to take advantage of the opportunities presented by the pandemic regarding entrepreneurship and successfully guiding them to take full advantage of these opportunities and, thus, contribute to the maintenance of social order and the enhancement of economic vitality is a question worthy of our attention. Based on event system theory, regulatory focus theory (RFT), and affect-as-information theory, this article analyzes the impact of COVID-19 on college students’ regulatory focus and fear of failure and then discusses its role on college students’ willingness to start their own businesses. Based on event system theory, for the role of the pandemic as an external factor, this article introduces the attribute of event strength to measure the impact of the event on the entity (Morgeson et al., 2015), i.e., to analyze the direct and indirect effects of the strength of the pandemic event on college students’ regulatory focus, fear of failure, and entrepreneurial intentions. RFT suggests that individuals possess two self-regulatory systems, promotion and prevention, which explain their behavioral motivations in depth; this theory was later applied in the field of entrepreneurship to provide a stronger explanation for entrepreneurial intentions (Higgins, 1997). In addition, affect-as-information theory suggests that an individual’s response to a specific event in the external environment is expressed as emotions, while negative emotions arising from the potential threat of failure in entrepreneurship are reflected in the fear of failure (Welpe et al., 2012). When individuals perceive the possibility of failure, changes in their behavior may follow (Urbano et al., 2016). As people increasingly form experiences about pandemics through pandemic-related information, the infectiousness of the emotions and the perceived fear associated with such crises may influence their intentions and even their behaviors (Ratten and Jones, 2021). In turn, fear of failure is an inherent part of the entrepreneurial journey, a product of the experience of entrepreneurs or potential entrepreneurs in ambiguous entrepreneurial context, and is key to explaining individuals’ choice to start a business or not (Morgan and Sisak, 2015; Cacciotti et al., 2020). Therefore, the fear of failure can be used as a bridge to explore the impact of the pandemic on the entrepreneurial intentions of college students. In summary, this article integrates event system theory, RFT, and affect-as-information theory to investigate the effect of event strength of COVID-19 pandemic on college students’ entrepreneurial intentions, alongside the mediating roles played by promotion regulatory focus and fear of failure and prevention regulatory focus and fear of failure, respectively, in order to enrich the relevant theoretical research and provide a framework for college students’ entrepreneurship during COVID-19 and post-COVID-19 eras.

The article will proceed as follows. First, we present six hypotheses based on a brief review of the literature related to event strength, regulatory focus, fear of failure, and entrepreneurial intentions. These relate to the effects of event strength on entrepreneurial intentions, the mediating role of fear of failure, and prevention and promotion regulatory focus on the relationship between them. We then describe the data collection, study measures, and sample characteristics of the data recovered. Finally, we report the empirical findings and describe the implications of this study.

## Theoretical Foundations and Research Hypotheses

### Relationship Between COVID-19 Pandemic and College Students’ Entrepreneurial Intentions

Event system theory dynamically examines the impact of events themselves and their attributes on individuals and organizations and mostly discusses them in three dimensions: strength, time, and space, where the event strength attributes include novelty, criticality, and disruptiveness (Morgeson et al., 2015). When considering the impact of an event on an entity, the effects of the three attributes need to be considered comprehensively. When the strength of an event is certain, the event that occurs at a time that meets the needs of the entity (timing) and continues (duration), originates at a higher level (origin), has a wider ripple effect (horizontal spread range), involves more levels (vertical spread range), and is closer to the entity (distance from the entity) is more likely to have an impact on the entity or to have a higher degree of impact. COVID-19 epidemic is a passive event and as of April 6, 2020, there are more than 131.4 million confirmed cases of COVID-19 worldwide, which still continues to grow. In terms of time attributes, the event is detrimental to the development of the entity and has a higher negative impact. In terms of space attributes, the epidemic has swept the globe since its outbreak, without exception and there is no difference in the relationship between entities and the event. Therefore, when discussing the impact of COVID-19 epidemic on entities, the impact of differences in the perceived intensity of entities is mainly examined.

For college students, entrepreneurship is often motivated by personal pursuits, interest, and attitudes toward entrepreneurship and policy support for entrepreneurship is also an incentive for them to choose entrepreneurship as a career (Mensah et al., 2021). However, the economic recession and recovery and the rise of some industries triggered by the unexpected event of COVID-19 have created more uncertainty about their career choices. Peter Drucker proposed that there are seven main sources of opportunity and the top two are unexpected success and unexpected failure, which means that opportunities mainly come from accidents. The massive outbreak of COVID-19 was clearly unexpected, causing a huge impact on all the industries while also disrupting the current business model and companies and individuals are in dire need of transformation under the crisis. The problem of rising unemployment caused by the spread of the pandemic is serious and encouraging young people to engage in entrepreneurship can reduce unemployment, promote the country’s economic development, and make the country and society more aware of the value of entrepreneurship, thus to provide more support for college students to start their own businesses such as supportive policies and entrepreneurship education (Lopes et al., 2021; Zhang and Huang, 2021; Zulfiqar et al., 2021). Except that, after several months of development, despite the slowdown in economic growth and the serious downward trend in some countries, we found that the non-contact economy is developing rapidly, for instance, online education, internet healthcare, and livestream shopping have achieved remarkable results, which to a certain extent show that the epidemic has given rise to entrepreneurial opportunities. At the same time, the epidemic also brings new problems to people’s working life such as daily protection, sterilization, transportation, and commuting. When individuals expand measures to solve the problems they face and help others to solve similar problems, they then develop into new businesses, i.e., user start-ups. Thus, we believe that the epidemic contains entrepreneurial opportunities that some individuals can identify and act upon. Economical factors can influence entrepreneurial intentions (Devece et al., 2016) and in a recession, there is a need to encourage the creation of new ventures by promoting identification and exploitation of entrepreneurial opportunities, thus stimulating economic dynamism (Ruiz-Rosa et al., 2020). The reduction in employment opportunities during the crisis reduces people’s access to income, thereby reducing the opportunity cost of creating new ventures and giving people greater incentive to use entrepreneurship as an alternative to being employed (Simón-Moya et al., 2016). At the same time, related research found that by creating, expanding, and replacing demand, COVID-19 is creating a wealth of entrepreneurial opportunities (Maritz et al., 2020), thereby creating rich prerequisites for college students to start their own businesses. When an individual perceives an entrepreneurial opportunity, he or she may choose to create a business (Karimi et al., 2016). In addition, the pandemic negatively affects small- and medium-sized enterprises, causing massive unemployment (Ratten and Jones, 2021). This may put considerable pressure on college students, especially those who are about to graduate, to find employment. Thus, for college students, the employment pressure and entrepreneurial opportunities created by COVID-19 pandemic may enhance their willingness to start their own businesses. Accordingly, we hypothesize that:

H1: The event strength of COVID-19 is positively associated with college students’ entrepreneurial intentions.

### Mediating Role of Fear of Failure

Fear of failure involves the fearful emotions caused by an individual’s perceived expectation of environmental threats to the achievement of his or her goals. When COVID-19 arrived, there was a general lack of awareness, the anxiety and fear among the public was spreaded, while the number of infections and deaths reported each day increased. Especially, during home isolation, individual college students were separated from the group and had more time to spend alone or with their families. The way people live and learn has changed dramatically, from face-to-face to online, and the spatial isolation makes people doubt the authenticity and richness of information, their psychological resources were severely depleted, their sense of control over the outside world and themselves were weaken, and generally lack a sense of security. They are still cautiously exposed even after the resumption of work and business and their daily life is gradually restored to order. After experiencing this epidemic, college students have a deep understanding of the uncertainty of the environment and are more uncertain about their future career development planning, showing a tendency to avoid it. College students are in the transition stage from school to society, full of confusion about their future, and fear increases when they actually feel the hardship of survival. According to the World Economic Outlook released by the International Monetary Fund (IMF), the outlook for global growth remains highly uncertain and will depend largely on developments in the epidemic and the effectiveness of policy actions in 2021. Even in China, which is showing positive growth, its economic growth rate is still lower than before and the downward pressure on the economy has increased, many catering and entertainment enterprises have gone bankrupt and closed down, and most of them are struggling to survive, so it has become a common initiative to reduce staff and salary. The possibility of unemployment upon graduation has increased their fear of the unknown and in this case, they are in urgent need of an alternative, either going onto higher education or starting their own business. In contrast, entrepreneurship is more feasible, especially with the popularization of innovation and entrepreneurship education, college students are more aware of entrepreneurship itself and have acquired certain thinking and methods to engage in entrepreneurial activities and the fear of failure in entrepreneurship is not unacceptable, although it is there. Studies have shown that economic crises and high unemployment potentially reducing employment opportunities and resulting in individuals losing their jobs or opportunities to pursue other careers, while also lowering their opportunity costs and making them more likely to choose entrepreneurship (Dawson and Henley, 2012; Simón-Moya et al., 2016). Moreover, Morris et al. (2020) found that entrepreneurship is promising for individuals with limited economic circumstances, with the simple creation and maintenance of a business demonstrating the potential to provide a better life. In this context, for individuals, especially college students facing employment, the difficulty of finding employment and the emergence of entrepreneurial opportunities may reduce the perceived risk of entrepreneurship compared with employment, making it more likely for them to set aside their fear of failure in order to choose entrepreneurship. On this basis, we hypothesize that:

H2: The event strength of COVID-19 pandemic can positively influence college students’ entrepreneurial intentions through the mediation of fear of failure.

### Mediating Role of Regulatory Focus

Regulatory focus theory explains the reasons, achieve ways and paths individuals exhibit different convergence or avoidance strategies when facing the same goal and is divided into prevention and promotion orientations according to the type of needs served (Higgins, 1997). The former is oriented to growth needs, focuses on self-actualization, pursues success, and adopts a convergence strategy; the latter is oriented to security needs, focuses on fulfilling obligations, emphasizes avoiding failure, and adopts an avoidance strategy. Crowe and Higgins (1997) suggested that both the promotion- and prevention-oriented regulatory systems exist in individuals, with trait tendencies or specific contexts determining their dominance. Under COVID-19 pandemic, downward pressure on the economy has increased, business operations are generally difficult, and salary reductions have become a common measure. The employment pressure of college students has increased and become more difficult, bargaining costs have increased, and competition has become more intense. In particular, when faced with an employer enterprise whose own operations are difficult and whose future is uncertain, the risks and costs of choosing to work for that enterprise are not necessarily significantly lower than those of self-employment and at the same time, the freedom and potential benefits that come with self-employment are lost. Khoa et al. (2020) suggested that most individuals are more vigilant when they recognize that the situation they are facing may have negative outcomes such as greater losses and tend to take other means to prevent it. Today’s college students, called Generation Z, care about their own experiences and when they find a certain experience better, they actively promote it to their surrounding friends and classmates, while constantly trying new things and working to uncover the best values and services. While COVID-19 pandemic is still ongoing, the vaccine is not yet widely injected and the means of prevention and control are mainly prevention and isolation. Although the anxiety of the public has been eased, there is still a sense of insecurity. With prevention regulatory focus, individuals seek safety, avoid losses, and tend to live and work in a safer and healthier way. Entrepreneurship has a higher degree of work flexibility and freedom, which can avoid the insecurity brought by frequent exposure and the inability to choose autonomously.

Trait theory suggests that a positive, proactive personality that facilitates opportunity exploration and problem solving in situations of crisis and uncertainty is an important factor influencing entrepreneurial intentions, which may be enhanced, if individuals hold positive expectations about future outcomes (Hernández-Sánchez et al., 2020). According to Escamilla-Fajardo et al. (2020) and others, the best way to deal with the opportunities presented by crises is to be proactive in seizing them to fill market gaps and that while COVID-19 epidemic poses many problems and challenges, the solution to the problem may well be where the opportunity lies. As we know, China’s internet economy is growing rapidly and e-commerce companies represented by Alibaba and Jingdong, in particular, are dominating the market. Looking back at the history of the two companies, we can see that it was the severe acute respiratory syndrome (SARS) outbreak in 2003 that gave rise to the transformation of the two companies, which led to the takeoff of e-commerce in China. What new industries and business models will emerge from COVID-19 epidemic and what impact it will have on the future are not yet predictable, but the empirical data from China show that online education and livestream shopping are favored by consumers and are performing remarkably well. College students follow the trends of the times, have a good grasp of the trends, are willing to try new things, and are able to clearly articulate their needs and actively seek solutions. With the promotion regulatory focus, individuals emphasize self-actualization and when they find that their demands are not fully satisfied or they can provide better solutions, they are willing to put in efforts and expect to gain benefits. In particular, as the pandemic progresses, the leading firms have made a shift and produced positive results, a phenomenon that greatly boosts individuals’ confidence that it is feasible to imitate or improve this business model. Accordingly, we propose the hypothesis that:

H3a: The event strength of COVID-19 pandemic can positively influence college students’ entrepreneurial intentions through the mediation of prevention regulatory focus.

H3b: The event strength of COVID-19 pandemic can positively influence college students’ entrepreneurial intentions through the mediation of promotion regulatory focus.

### Continuous Mediation Role of Fear of Failure and Regulatory Focus

Hernández-Sánchez et al. (2020) pointed out that psychological, cognitive, and personality variables contribute to the development of entrepreneurial intentions in uncertain or risky environments and this article found that fear of failure and regulatory orientation play an intermediate role. COVID-19 epidemic stimulated feelings of anxiety and insecurity of public and in the face of employment, the college student groups generally exhibited fear of failure. When facing potential risks, individuals differ in their tendency to be autonomous about the event and their willingness to influence the environment and fear of failure relates to the individual’s tendency to be stable in influencing their behavior and environment. This generates shame about the possibility of potential failure, requiring the expenditure of cognitive resources to regulate negative emotions (Hirst et al., 2020). In order to achieve the imperative task of employment, college students need to engage in self-regulation, which can be specifically categorized as prevention and promotion orientations. According to Leder et al. (2013), when thinking about the likelihood of making poor decisions, individuals with both the prevention and promotion regulatory focus should focus on avoiding negative outcomes, where it is more likely for individuals with prevention regulatory focus to worry about potential losses and more likely for individuals with promotion regulatory focus to worry about no gains. College students with prevention regulatory focus are willing to take responsibility and see employment as something they must accomplish at the end of their college career, which are responsible for themselves, for their families, and for society. Simón-Moya et al. (2016) argued that the survival rate of new ventures is instead higher in times of economic crisis than usual. Moreover, they argued that during economic downturns, due to the increased scarcity of paid work and the lower opportunity cost of entrepreneurship, the risk of individuals choosing entrepreneurship is not necessarily higher than the risk of unemployment and individuals may be more willing to engage in entrepreneurship rather than risking a high unemployment rate to find a job. When the risk of unemployment is elevated and the risk of entrepreneurship is relatively lower, individuals tend to avoid risk and entrepreneurial intentions are elevated under prevention regulatory focus. Most businesses have difficulty in surviving under the epidemic, but it is not difficult to find that the digital economy is developing strongly, especially online education and platform live streaming, which are relatively low threshold for entrepreneurship and are accessible and well understood by college students on a daily basis. Under promotion regulatory focus domination, individuals focus on earnings and success, college students value more the “opportunity” in danger and although they fear failure, they noticed that there are already successful examples, which can be imitated and realized. The level of fear of failure is higher under the epidemic, but compared with employment, entrepreneurship can bring more benefits and sense of achievement to individuals and considering individuals’ desire for success, individuals’ willingness to start a business increased under the promotion regulatory focus. Accordingly, we propose the hypothesis that:

H4a: The event strength of COVID-19 pandemic can positively influence college students’ entrepreneurial intentions through the continuous mediation of fear of failure and prevention regulatory focus.

H4b: The event strength of COVID-19 pandemic can positively influence college students’ entrepreneurial intentions through the continuous mediation of fear of failure and promotion regulatory focus.

## Data and Research Methodology

### Data Collection

In order to test the hypothesis, we collected data through questionnaires. In this article, we study the impact of the pandemic on college students’ entrepreneurial intention and the respondents are college students. According to the Chinese Professionals and Entrepreneurs Association (CPEA) index, we randomly selected college students as respondents in six universities in Guangzhou, Tianjin, Harbin, and Changchun and invited them to complete the questionnaire. Prior to data collection, participants were assured that the details they provided would be confidential and that the survey would only be continued with their consent. After that, we started the data collection process, using two main steps to collect data. The first was a face-to-face interview to enable the respondents to understand the purpose of this study. Then, a total of 282 questionnaires were completed and the data collection process took 3 months, from June 2020 to September 2020. However, in order to ensure high quality and validity of the questionnaires, 37 invalid questionnaires that were either interrupted during the filling process were filled out in too short a time (less than 5 min) or contained gaps or invalid information were eliminated. Finally, we obtained 245 valid questionnaires with high quality and validity. The sample characteristics are shown in Table 1.

**TABLE 1:** Sample characteristics (*N* = 245).

Variables	Items	Frequency	Proportion (%)	Variables	Items	Frequency	Proportion (%)
Gender	Male	153	62.4	Region	Northeast	8	3.3
	Female	92	37.6		Central	45	18.4
Participation in entrepreneurship competitions	Yes	108	44.1		Eastern	157	64.1
	No	137	55.9		Western	32	13.1
Work experience	Yes	138	56.3		Other	3	1.2
	No	107	43.7				

### Variable Measurement

(1) Event strength: Based on Morgeson et al.’s (2015) research, this study measured the strength of COVID-19 pandemic using 11 items, including “the approach to COVID-19 pandemic is clear and understandable.”
(2) Fear of failure: This study measured the fear of failure based on the Kollmann et al. (2017) scale using 5 items, including “I am afraid of failing in difficult situations when a lot of things depend on me.”
(3) Prevention regulatory focus: Based on Neubert et al.’s (2008) research, 9 items, such as “I focus on completing the task correctly to increase job security,” were used to measure prevention regulatory focus.
(4) Promotion regulatory focus: Based on Neubert et al.’s (2008) research, 9 items, such as “I take advantage of opportunities at work to maximize my goals for advancement,” were used to measure promotion regulatory focus.
(5) Entrepreneurial intention: Based on Piperopoulos and Dimov (2014), the question “How likely are you to start your own company in the future?” was used to measure entrepreneurial intention.
(6) Control variables: Control variables are variables other than independent variables that affect the outcome. In this empirical study, we used gender, region, participation in entrepreneurial competitions, and participation in entrepreneurial courses as the control variables.

### Reliability and Validity Tests

In order to ensure that the selected measurement method could be effectively used in subsequent empirical studies, this study used a presurvey to test the reliability and validity of the questionnaire, with a total of 114 valid questionnaires collected during the presurvey stage.

(1) Reliability: The Cronbach’s alpha values of the constructs were used in this study to test the reliability of the scale. As shown in Table 2, the Cronbach’s alpha values of the variables involved in this study were all above the threshold of 0.700 and the composite reliability (CR) was also above the critical value of 0.700, indicating that the scale in this study possessed a high level of reliability.

**TABLE 2:** Reliability and validity.

Item	Factor loading	Item	Factor loading
Prevention regulatory focus (α = 0.957, CR = 0.9585, AVE = 0.7201)		Event strength (α = 0.930, CR = 0.9223, AVE = 0.5204)	
PRE1	0.909	XG1	0.779
PRE2	0.910	XG2	0.815
PRE3	0.814	XG3	0.682
PRE4	0.825	XG4	0.750
PRE5	0.823	XG5	0.715
PRE6	0.848	XG6	0.722
PRE7	0.847	XG7	0.722
PRE8	0.799	XG8	0.744
PRE9	0.855	XG9	0.637
Promotion regulatory focus (α = 0.930, CR = 0.9333, AVE = 0.6094)		XG10	0.635
		XG11	0.714
PRO1	0.870	Fear of failure (α = 0.930, CR = 0.8878, AVE = 0.6172)	
PRO2	0.813		
PRO3	0.760	FF1	0.988
PRO4	0.756	FF2	0.718
PRO5	0.786	FF3	0.709
PRO6	0.745	FF4	0.773
PRO7	0.730	FF5	0.703
PRO8	0.803		
PRO9	0.753		

*χ^2^/df = 1.184, IFI = 0.964, TLI = 0.961, CFI = 0.964, RMSEA = 0.040.*

*XG, event strength; PRE, prevention regulatory focus; PRO, promotion regulatory focus; FF, fear of failure.*

(2) Validity: For mature scales, the validity level can be tested directly by confirmatory factor analysis (CFA). The AMOS version 21.0 was used in this study for CFAs, which showed that all the indices were within good range, where c2/df (<3), comparative fit index (CFI) (>0.900), root mean square error of approximation (RMSEA) (<0.08) and the model fit were good, as seen in Table 1. The standardized regression weights of each question item for the variables exceeded the critical level of 0.500 and the average variance extracted (AVE) values of all the variables exceeded the critical value of 0.500, with good convergent validity. In addition, the scale is considered to have good discriminative validity when the square root of the AVE value of the variable is greater than the absolute value of its correlation coefficient with other variables. As indicated by the correlation matrix in Table 3, the square roots of AVE on the diagonal were greater than the absolute value of the correlation coefficients.

**TABLE 3:** Correlations, means, and SDs.

Variable	1	2	3	4	5	6	7	8	9
1 Gender									
2 Region	−0.138*								
3 Entrepreneurial competitions	–0.008	0.046							
4 Entrepreneurship courses	–0.020	0.024	0.003						
5 XG	–0.016	0.005	0.022	–0.097	**0.849**				
6 FF	0.088	0.101	–0.078	0.055	0.133*	**0.786**			
7 PRE	0.003	0.008	–0.046	0.028	0.142*	0.470**	**0.781**		
8 PRO	0.023	0.013	–0.077	0.117	0.183**	0.562**	0.687**	**0.721**	
9 EI	0.075	0.045	–0.048	0.099	0.116	0.619**	0.527**	0.635**	**—**
Mean	1.376	2.906	1.559	1.437	4.188	4.646	5.115	5.458	4.674
S.D.	0.485	0.698	0.498	0.497	0.484	1.189	0.572	0.822	1.248

*Indicates p < 0.05 (two-tailed test), **indicates p < 0.01 (two-tailed test), and n = 245.

*Data below the diagonal indicate correlation coefficients, and bolded data on the diagonal are the square roots of AVE.*

*EI in the table indicates entrepreneurial intention.*

### Common Method Bias

This study used Harman’s single factor test to test for common method bias, using principal component analysis to analyze the items of all the variables to obtain the unrotated factor variance. The results showed that the first principal component factor explained 27.554% of the total variance, which was below the threshold of 40% and did not explain most of the variance. This indicated that the research data in this study did not have a serious common method bias problem and that the impact caused by the subsequent analysis was not significant.

## Results

### Descriptive Statistics and Correlation Analysis

The means, SDs, and correlation coefficients among the variables involved in this study are shown in Table 3. The results of the descriptive statistics and correlation analysis showed that the means and SDs of the variables were basically within a reasonable range and the correlation coefficients between the main variables were basically unexceptional and correlated.

### Hypothesis Testing

(1) Direct effect test: This study used the SPSS version 22.0 to test the hypotheses proposed in this article by hierarchical regression, with the results shown in Table 4. Model 1 was the regression model of control variables on fear of failure, model 2 was the regression model of control variables and event strength on fear of failure, model 3 was the regression model of control variables on prevention regulatory focus, model 4 was the regression model of control variables and event strength on prevention regulatory focus, model 5 was the regression model of control variables, event strength, and fear of failure on prevention regulatory focus, model 6 was the regression model of control variables on entrepreneurial intentions, model 7 was the regression model of control variables and event strength on entrepreneurial intentions, and model 8 was for control variables, event strength, fear of failure, and prevention regulatory focus on entrepreneurial intentions. Model 7 showed that there was a significant positive effect of event strength on entrepreneurial intentions (β = 0.129, *p* < 0.05), indicating a positive relationship between event strength and entrepreneurial intentions, thereby proving H1. Model 2 showed a significant positive relationship between event strength and fear of failure (β = 0.142, *p* < 0.05), whereas the results of model 5 showed a significant positive relationship between fear of failure and prevention regulatory focus (β = 0.466, *p* < 0.001). The results of model 8 showed significant positive relationship between both the fear of failure and prevention regulatory focus with entrepreneurial intentions, respectively (β = 0.468, *p* < 0.001; β = 0.303, *p* < 0.001).

**TABLE 4:** Regression analysis results (PRE path).

Variables	Fear of failure		Prevention regulatory focus			Entrepreneurial intentions		
	Model 1	Model 2	Model 3	Model 4	Model 5	Model 6	Model 7	Model 8
**Control variables**								
Genders	0.105	0.107	0.005	0.007	–0.043	0.084	0.086	0.034
Region	0.118	0.117	0.010	0.009	–0.046	0.056	0.056	–0.002
Entrepreneurial competition	–0.083	–0.086	–0.046	–0.049	–0.009	–0.050	–0.053	0.002
Entrepreneurial courses	0.054	0.068	0.028	0.042	0.011	0.099	0.112	0.067
XG		0.142*		0.147*	0.081		0.129*	0.018
FF					0.466***			0.468***
PRE								0.303***
R2	0.031	0.051	0.003	0.024	0.231	0.021	0.037	0.460
Adjust R2	0.014	0.031	–0.014	0.004	0.212	0.005	0.017	0.444
F-value	1.891	2.548*	0.179	1.199	11.915***	1.286	1.857	28.856***

*Data in the table are standardized coefficients.*

*Indicates p < 0.05, ***indicates p < 0.001.

For the promotion regulatory focus, model 9 was the regression model of control variables on fear of failure, model 10 was the regression model of control variables and event strength on fear of failure, model 11 was the regression model of control variables on promotion regulatory focus, model 12 was the regression model of control variables and event strength on promotion regulatory focus, model 13 was the regression model of control variables, event strength, and fear of failure on promotion regulatory focus, model 14 was the regression model of control variables on entrepreneurial intentions, model 15 was the regression model of control variables and event strength on entrepreneurial intentions, and model 16 was for control variables, event strength, fear of failure, and promotion regulatory focus on entrepreneurial intentions. The results are shown in Table 5. Model 13 showed a significant positive relationship between fear of failure and promotion regulatory focus (β = 0.545, *p* < 0.001). The results of model 16 showed significant positive relationship between both the fear of failure and promotion regulatory focus with entrepreneurial intentions, respectively (β = 0.381, *p* < 0.001; β = 0.419, *p* < 0.001).

**TABLE 5:** Regression analysis results (PRO path).

Variables	Fear of failure		Promotion regulatory focus			Entrepreneurial intentions		
	Model 9	Model10	Model11	Model12	Model13	Model14	Model15	Model16
**Control variables**								
Genders	0.105	0.107	0.027	0.031	–0.028	0.084	0.086	0.033
Region	0.118	0.117	0.018	0.017	–0.047	0.056	0.056	0.004
Entrepreneurial competition	–0.083	–0.086	–0.078	–0.082	–0.036	–0.050	–0.053	0.014
Entrepreneurial courses	0.054	0.068	0.118	0.137	0.100	0.099	0.112	0.028
XG		0.142*		0.198**	0.121*		0.129*	–0.008
FF					0.545***			0.381***
PRO								0.419***
R2	0.031	0.051	0.021	0.060	0.342	0.021	0.037	0.505
Adjust R2	0.014	0.031	0.004	0.040	0.325	0.005	0.017	0.491
F-value	1.891	2.548*	1.267	3.026*	20.575***	1.286	1.857	34.601***

*Data in the table are standardized coefficients.*

*Indicates p < 0.05, **indicates p < 0.01, ***indicates p < 0.001.

(2) Considering the relatively low statistical power of the stepwise method, it is easy to produce type I error (Preacher and Hayes, 2008). Therefore, this study adopted the SPSS macroinstruction “PROCESS” to conduct the mediation test through the bootstrap method. The sampling was repeated 5,000 times with 95% CIs to ensure more robust results, which are shown in Table 6. The results showed that event strength affects entrepreneurial intentions through the following paths: event strength→fear of failure→entrepreneurial intentions (0.1716), event strength→fear of failure→prevention regulatory focus→entrepreneurial intentions (0.0518), event strength→fear of failure→entrepreneurial intentions (0.1396), event strength→promotion regulatory focus→entrepreneurial intentions (0.1303), and event strength→fear of failure→promotion regulatory focus→entrepreneurial intentions (0.0838). The path of event strength→prevention regulatory focus→entrepreneurial intentions, on the other hand, showed insignificance. Hypotheses H2, H3b, H4a, and H4b were supported, whereas hypothesis H3a was not supported.

**TABLE 6:** Bootstrap mediation effect test results.

Hypotheses	Coefficient	Standard error	LLCI	ULCI
Total indirect effects	0.2866	0.1048	0.0781	0.4950
XG→FF→EI	0.1716	0.0816	0.0178	0.3397
XG→PRE→EI	0.0632	0.0479	-0.0314	0.1597
XG→FF→PRE→EI	0.0518	0.0272	0.0054	0.1127
Total indirect effects	0.3537	0.1174	0.1317	0.5937
XG→FF→EI	0.1396	0.0709	0.0113	0.2915
XG→PRO→EI	0.1303	0.0600	0.0240	0.2596
XG→FF→PRO→EI	0.0838	0.0494	0.0053	0.2007

## Conclusion and Discussion

### Findings and Discussion

On the basis of event system theory, RFT, and affect-as-information theory, this article explored the influencing mechanism of the event strength of COVID-19 on college students’ entrepreneurial intentions and investigated the mediating role of regulatory focus and fear of failure in this relationship. Combining the theoretical research with the empirical study of data obtained *via* questionnaires, we found that the event strength of COVID-19 pandemic has a positive effect on college students’ entrepreneurial intentions and this relationship can be transmitted through the continuous mediating role of regulatory focus and fear of failure.

However, the empirical results showed that hypothesis H3 was not proven, i.e., the fear of entrepreneurial failure did not play a significant role in mediating the relationship between the event strength of COVID-19 and college students’ entrepreneurial intentions. This suggests that the event strength of COVID-19 cannot affect entrepreneurial intentions through fear of failure without considering the role of regulatory focus. This may due to the fact that, from an agentic perspective, self-regulation is required for individuals to cope with dynamic changes in the environment by consuming limited cognitive resource capacity. Locking in a relatively static future through fear of failure alone may make it difficult to adapt to rapid changes and form effective strategies, thereby further increasing cognitive resource consumption to cope with negative emotions such as fear of failure (Neal et al., 2017; Hirst et al., 2020). Thus, it may be difficult to adequately transmit the impact of the dynamic changes brought about by COVID-19 pandemic on students’ entrepreneurial intentions through fear of failure alone.

### Research Contributions

First, COVID-19 pandemic continues to be a worldwide emergency, with a broad impact on the global economic environment. While research explored the impact of major events such as natural disasters, pandemics, and financial crises, COVID-19 pandemic, unlike previous events, is a long-running, persistent public health crisis causing significant changes to people’s lifestyles on multiple levels (Zahra, 2021). In the field of management, particularly in entrepreneurship, research has not yet had time to fully expand. This study addresses its impact on the college students’ entrepreneurial intentions in order to explore the willingness of college students affected by the pandemic to take advantage of entrepreneurial opportunities and the theoretical underpinnings of these choices, thereby enriching the theoretical findings in this field.

Second, Hernández-Sánchez et al. (2020) suggested that studying the willingness of pandemic-affected students (potential entrepreneurs) to start a business needs to be approached in terms of personality. Regulatory focus provides insight into the motivation of individuals to engage in behaviors. This article began with the RFT to investigate whether the pandemic-affected college students were willing to engage in entrepreneurship, demonstrating that, under the employment difficulties and opportunities brought about by COVID-19, prevention regulatory focus and promotion regulatory focus made college students less willing to be employed and more willing to start a business. In previous studies, the roles of the two regulatory focuses were often shown to be contradictory, but in the pandemic crisis, the roles of the two regulatory focuses on college students’ willingness to start their own businesses were finally unified from different directions. From this point of view, the evidence presented herein may help to draw some new conclusions.

Finally, in practice, the research results in this article help college students facing employment to better understand the impact of COVID-19 on current employment and market opportunities and also lead them to fully self-analyze when they face the decision of whether to start a business, so that during the pandemic and postpandemic eras, college students can be more rational when facing employment or entrepreneurship decisions. In addition, the results of this research are also useful for governments to provide more reasonable and effective information and guidance to college students and the general public. Therefore, these groups could make more rational choices and individuals with entrepreneurial potential and ideas could be guided to engage in entrepreneurial activities, thus contributing to stimulating economic activity and rebooting the economy.

### Limitations and Future Research

There are still some limitations present in this article. First, in this study, cross-sectional data were used because COVID-19 pandemic happened suddenly; it was difficult to fully estimate the severity of the matter at the beginning of the pandemic and the duration is not yet long enough to form a continuous follow-up of the affected college students, which may have affected the depth of this study. It is believed that, with continued follow-up of students affected by the pandemic, more in-depth study may be developed in the future.

Second, as the pandemic varies greatly from country to country around the world, the extent of its impact on economic development, employment, and other things is also variable. In order to ensure the availability of research data, this study only collected data from Chinese college students; therefore, the findings are more applicable to China’s national conditions; applicability to other countries may be reduced. Future research may choose college students from other countries as subjects or collect more extensive data to explore more generalizable research results.

Finally, this article analyzes the impact of COVID-19 pandemic on entrepreneurial intentions from the perspective of college students’ personality traits and emotions, but the factors affecting college students’ entrepreneurial intentions may be broader and future research could focus on the role of more variables such as entrepreneurship education and entrepreneurship policies.

## Data Availability Statement

The raw data supporting the conclusions of this article will be made available by the authors, without undue reservation.

## Ethics Statement

Ethical review and approval were not required for the study on human participants in accordance with the local legislation and institutional requirements. Written informed consent from the participants was not required to participate in this study in accordance with the national legislation and the institutional requirements.

## Author Contributions

FS: writing and processing data. YC: providing revised advice. Both authors contributed to the article and approved the submitted version of the manuscript.

## Conflict of Interest

YC was employed by the company Dalian Yongjia Electronic Technology Co., Ltd. The remaining author declares that the research was conducted in the absence of any commercial or financial relationships that could be construed as a potential conflict of interest.

## Publisher’s Note

All claims expressed in this article are solely those of the authors and do not necessarily represent those of their affiliated organizations, or those of the publisher, the editors and the reviewers. Any product that may be evaluated in this article, or claim that may be made by its manufacturer, is not guaranteed or endorsed by the publisher.
